# Changes in electrical delay is associated with the hemodynamic response to cardiac pacing

**DOI:** 10.1016/j.hroo.2024.03.013

**Published:** 2024-04-04

**Authors:** Stian Ross, Øyvind H. Lie, Hans H. Odland, Trine Fink, Thor Edvardsen, Kristina H. Haugaa, Erik Kongsgaard

**Affiliations:** ∗ Department of Cardiology, Oslo University Hospital, Rikshospitalet, Norway; † Institute for Clinical Medicine, University of Oslo, Norway

**Keywords:** Cardiac resynchronization therapy, Hemodynamic response, Electrical delay, Electrocardiography, Heart failure

**Key Findings**

▪ Time to onset of the steepest deflection measured with 2 electrocardiographic electrodes is a simple method for assessment of left ventricular electrical dyssynchrony.
▪ Reduction of ventricular electrical delay was a marker of favorable hemodynamic response to both right ventricular and biventricular pacing.
▪ Activation times routinely measured in conduction system pacing may provide valuable information also in conventional pacing.

Landmark studies of heart failure patients with prolonged QRS duration on electrocardiography (ECG) demonstrated that biventricular pacing (BIVP) was superior to right ventricular pacing (RVP).^1^ Detrimental long-term effects and unpredictable acute hemodynamic effects due to RVP led to the existing skepticism regarding RVP, and BIVP became favored as the standard of care implementing cardiac resynchronization therapy (CRT).^2^ However, despite reductions in mortality and morbidity, one-third of eligible patients do not benefit from BIVP, thus demonstrating the shortcoming of QRS duration and morphology in predicting CRT response.

Patients with left bundle branch block (LBBB) typically have deep S waves on ECG electrode V_1_ and large R waves on lead V_5_ representing a septal-to-lateral activation sequence. Plesinger et al^3^ reported that ventricular electrical delay may be assessed as the dispersion of peak septal and lateral ECG amplitudes, and that high ventricular electrical delay at sinus rhythm was associated with greater CRT benefit.

Conduction system pacing has recently become of great interest and left bundle branch area pacing in particular seems to be an attractive pacing option. Rapid activation is the overall aim of cardiac pacing and is measured continuously during the left bundle branch area pacing procedure. Jastrzebski et al^4^ recently showed that the V_6_–V_1_ interpeak interval is a novel criterion for LBB capture confirmation. The intrinsic conduction system is not used with RVP and BIVP. However, we hypothesized that measurements of cardiac activation times would provide valuable information also in conventional pacing.

In the present acute experimental study, we aimed to investigate the association between the electrical and hemodynamic changes induced by cardiac pacing and hypothesized that modification of the ventricular electrical delay caused by RVP and BIVP would influence the acute hemodynamic response in patients undergoing CRT implantation. The Regional Ethical Committee for Medical Research (REK South East, Oslo, Norway) approved the research protocol, and the study complied with the Declaration of Helsinki. All patients provided written informed consent. Statistical analysis was performed by linear mixed model regression (STATA SE 15.1, StataCorp, College Station, TX) Values are given mean (95% confidence interval).

**Figure 1:**
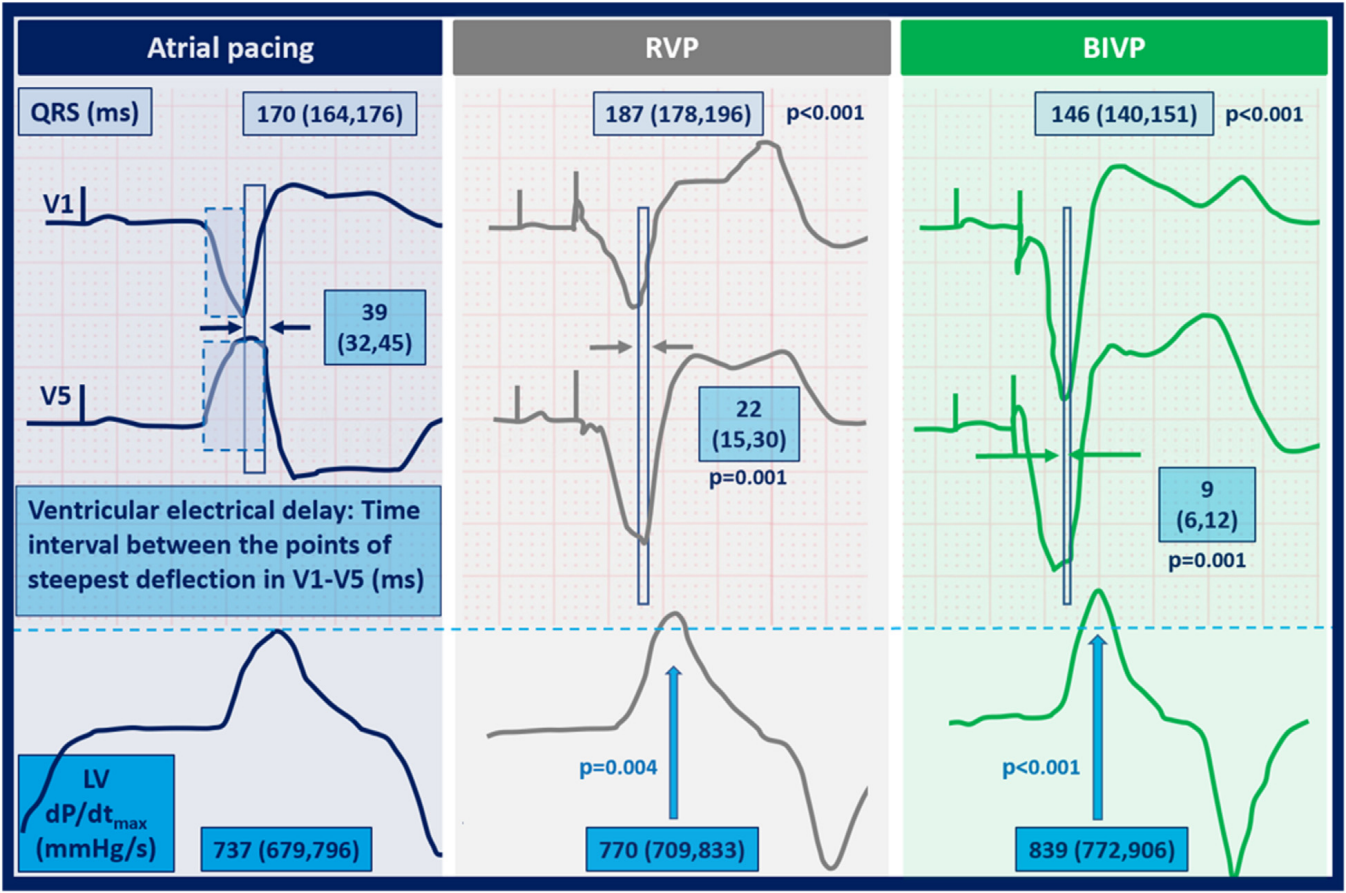
Electromechanical response to various forms of cardiac pacing. Panels represent electrical and hemodynamic response to pacing. Time from QRS onset to point of steepest deflection toward the isoelectric line was assessed in ECG electrodes V_1_ and V_5_ (*blue boxes with dotted line;***left**). Ventricular electrical delay was defined as the difference between these points. S waves were used in leads V_1_ and V_5_ in patients with paced negative concordant QRS vector. The increase in LV dP/dt_max_ was associated with a stepwise reduction in ventricular electrical delay caused by right ventricular pacing (RVP) **(middle)** and biventricular pacing (BIVP) **(right)** independent of the changes in QRS duration. dP/dt_max_ = maximum rate of left ventricular pressure change; LV = left ventricle.

Thirty-eight patients (age 63 ± 10 years; 31% women; 40% ischemic) with LBBB and mean ejection fraction of 29% ± 5% were included in the study. Atrial pacing was followed by sequential RVP and BIVP with patient-specific paced AV delay (142 ± 30 ms). Acute hemodynamic response was defined as the maximum rate of invasive left ventricular (LV) pressure change (LV dP/dt_max)_. ECG analyses were performed using digital software (LabChart Pro 8.0, ADInstruments Ltd., Oxford, United Kingdom) and averaged over 3 consecutive heart beats. Ventricular electrical delay was assessed using ECG electrodes V_1_ and V_5_. We measured the time from intrinsic QRS onset or pacing spike to the point of steepest deflection at or after the peak amplitude of the S wave or R wave (Figure 1, left). Ventricular electrical delay was defined as the absolute time difference between these 2 points.

We observed improved LV dP/dt_max_ with decreasing ventricular electrical delay independent of pacing site [10 (4–16) mm Hg/s increase in LV dP/dt_max_ per 10-ms reduction in ventricular electrical delay; *P* = .002]. Compared to baseline, the increase in LV dP/dt_max_ during RVP was 32 (10–54) mm Hg/s (*P* = .004), but this effect was confounded by a reduction in ventricular electrical delay [adjusted beta LV dP/dt_max_ 17 (–5 to 390; *P* = .14]. LV dP/dt_max_ increased 74 (46–101) mm Hg/s with BIVP (*P* <.001), plus an additional 10 mm Hg/s per 10-ms reduction in ventricular electrical delay. Our results were independent of paced QRS duration (Figure 1). This finding indicates that alignment of V_1_ and V_5_ reflects reduced LV electrical dyssynchrony regardless of paced QRS duration and translates into beneficial hemodynamics.

In line with previous data, we found a large variation in acute hemodynamic response to RVP, ranging from –21% to +20% change in LV dP/dt_max_ compared to atrial pacing. Ventricular electrical delay identified patients with LV dP/dt_max_ increase during RVP (area under the curve 0.78; *P* = .009). However, whether a reduction in ventricular electrical delay is indicative of the ability to withstand pacing-induced heart failure remains to be explored.

Time to onset of the steepest deflection measured in 2 precordial ECG electrodes is a simple method for assessment of LV electrical dyssynchrony in heart failure patients with LBBB. Reduction of ventricular electrical delay was a marker of favorable hemodynamic response to both RVP and BIVP and needs to be validated in future studies with a larger cohort and include determination of long-term response and clinical outcome.
